# Meta-Analysis of Genome-Wide Association Studies and Network Analysis-Based Integration with Gene Expression Data Identify New Suggestive Loci and Unravel a Wnt-Centric Network Associated with Dupuytren’s Disease

**DOI:** 10.1371/journal.pone.0158101

**Published:** 2016-07-28

**Authors:** Kerstin Becker, Sabine Siegert, Mohammad Reza Toliat, Juanjiangmeng Du, Ramona Casper, Guido H. Dolmans, Paul M. Werker, Sigrid Tinschert, Andre Franke, Christian Gieger, Konstantin Strauch, Michael Nothnagel, Peter Nürnberg, Hans Christian Hennies, German Dupuytren Study Group

**Affiliations:** 1 Cologne Center for Genomics, University of Cologne, Cologne, Germany; 2 Cluster of Excellence on Cellular Stress Responses in Aging-associated Diseases, University of Cologne, Cologne, Germany; 3 University of Groningen and University Medical Center Groningen, Dept. of Plastic Surgery, Groningen, the Netherlands; 4 Div. of Human Genetics and Dept. of Dermatology, Medical University of Innsbruck, Innsbruck, Austria; 5 Inst. of Clinical Genetics, Dresden University of Technology, Dresden, Germany; 6 Institute of Clinical Molecular Biology, Christian-Albrechts-University of Kiel, University Hospital Schleswig-Holstein, Kiel, Germany; 7 Research Unit Molecular Epidemiology, Helmholtz Zentrum München, Neuherberg, Germany; 8 Institute of Epidemiologie II, Helmholtz Zentrum München, Neuherberg, Germany; 9 German Center for Diabetes Research, Neuherberg, Germany; 10 Institute of Genetic Epidemiology, Helmholtz Zentrum München, Neuherberg, Germany; 11 Dept. of Biological Sciences, University of Huddersfield, Huddersfield, United Kingdom; Flinders University, AUSTRALIA

## Abstract

Dupuytren´s disease, a fibromatosis of the connective tissue in the palm, is a common complex disease with a strong genetic component. Up to date nine genetic loci have been found to be associated with the disease. Six of these loci contain genes that code for Wnt signalling proteins. In spite of this striking first insight into the genetic factors in Dupuytren´s disease, much of the inherited risk in Dupuytren´s disease still needs to be discovered. The already identified loci jointly explain ~1% of the heritability in this disease. To further elucidate the genetic basis of Dupuytren´s disease, we performed a genome-wide meta-analysis combining three genome-wide association study (GWAS) data sets, comprising 1,580 cases and 4,480 controls. We corroborated all nine previously identified loci, six of these with genome-wide significance (p-value < 5x10^-8^). In addition, we identified 14 new suggestive loci (p-value < 10^−5^). Intriguingly, several of these new loci contain genes associated with Wnt signalling and therefore represent excellent candidates for replication. Next, we compared whole-transcriptome data between patient- and control-derived tissue samples and found the Wnt/β-catenin pathway to be the top deregulated pathway in patient samples. We then conducted network and pathway analyses in order to identify protein networks that are enriched for genes highlighted in the GWAS meta-analysis and expression data sets. We found further evidence that the Wnt signalling pathways in conjunction with other pathways may play a critical role in Dupuytren´s disease.

## Introduction

Dupuytren’s disease (DD; OMIM 126900) is among the most common genetic diseases of connective tissues. DD is a fibroproliferative disorder that affects the palmar aponeurosis and the cutaneous retinacula of the hand. It manifests first through the formation of subcutaneous nodules, fibrotic cords are found afterwards, and flexion contractures of single fingers can then occur. There is no known cure for the disease. Surgical intervention is the most common treatment option when hand function becomes hampered. The prevalence of DD has been estimated as around 4% in England [1] and 2.5% in Germany [2]. It increases significantly with age [3, 4] and was determined as 22% in a cross-sectional study of the population aged over 50 years in the northern part of the Netherlands [5] and 30% in the Norwegian population over 60 years of age [6]. Histopathologically, an increased proliferation of fibroblasts and differentiation into myofibroblasts can be associated with a massive deposition of extracellular matrix (ECM). The disease shows a progressive clinical behaviour with frequent local recurrence.

The causes for DD are multifactorial. Several environmental factors have been proposed to contribute to DD development. Smoking and alcohol consumption have been associated with DD [7–9]. Elevated blood glucose levels, low body weight, and low body mass index (BMI) have also been shown to contribute to DD [9]. Heavy manual labour and exposure to vibrations probably trigger the manifestation of the disease [10, 11]. Importantly, DD has a strong genetic basis. Frequent familial occurrence is observed [12]. Studies have determined a family predisposition in 12.5% [2] and 27% [13] of cases, respectively. The sibling recurrence risk λ_s_ has been estimated to equal 2.9 based on a prevalence of 3.5% in northwestern England [14]. Patients with known family history show a trend to undergo surgery earlier than patients without known family history [15]. DD has a polygenic basis, where different genetic risk loci strongly contribute to disease susceptibility [16]. In a recent population-based twin study the heritability of DD was calculated to be 80% [17].

The first GWAS for this disease [18] identified nine genome-wide significant loci. Six of these loci contain genes known to be involved in Wnt signalling, namely *WNT4*, *SFRP4*, *WNT2*, *SULF1*, *RSPO2*, and *WNT7B*. Notably, these six genes are either Wnt ligands or extracellular modulators of Wnt signalling, and therefore lie upstream of the Wnt signalling cascade. This finding was the first insight that Wnt signalling may be one of the molecular mechanisms that underlie the genetics of DD. The Wnt signalling pathway plays an important role during embryogenesis and is tightly regulated on multiple levels [19]. Considering the late age of onset in DD, which peaks in the 5^th^ decade of life [5, 15], highly damaging mutations in Wnt or other genes are not expected in DD. Accordingly, the majority of SNPs associated with complex diseases map to non-coding regions, and it has been suggested that regulatory effects commonly underlie these GWAS signals [20]. Many of these regulatory effects will be small and difficult to detect individually [21]. On top of that, GWAS for complex diseases usually point to only a proportion of the causal genetic basis of the respective diseases and many signals attributed to causal genetic variants may be hidden in the statistical noise of those GWAS. A systems approach to analyse the pathways and networks involved in DD may overcome at least partially these limitations. Network modelling can help to increase the statistical power to detect susceptibility loci with more subtle effects by including candidate loci and modeling relationships between these loci and previously known, significantly associated loci derived from GWAS. Network modelling is used extensively for gene expression data analysis, and a consensus network interfered from mutable methods was shown to perform best [22].

To identify new loci associated with DD and corroborate the nine previously described GWAS loci we performed a meta-analysis on the imputed genotypes of three genome-wide GWAS data sets comprising 1,580 cases and 4,480 controls. As a first step in a systems genetics approach to understand the pathogenesis of DD, we have integrated the GWAS results with whole-transcriptome data. In a network analysis based on protein-protein interaction data we identified functional modules of genes/proteins with shared characteristics that were overrepresented in our DD case/control data sets.

## Methods

### Study participants

The study was approved by the Ethics Commission of the Faculty of Medicine of the University of Cologne and participants provided written informed consent. All participating subjects were of European origin (see S1 and S3 Tables for details). The German study population is described in detail elsewhere [15]. DNA was extracted from peripheral blood samples. RNA was obtained from primary tissue samples from patients and sex and age matched controls. Control connective tissue samples were obtained during trigger finger or carpal tunnel release.

### Data sets

We included 1,580 cases and 4,480 controls in a meta-analysis on the imputed genotypes of three genome-wide GWAS data sets (S1 Table). Samples were genotyped on three different genotyping platforms. The first data set comprised 186 cases from Germany and Switzerland typed on the Affymetrix Genome-Wide Human SNP Array 6.0 (809,858 SNPs) and 447 controls typed on the same array from the KORA study (KORA [Cooperative Health Research in the Region of Augsburg], Helmholtz Center Munich). The second data set consisted of 538 German and Swiss cases and 1197 controls from the Popgen study (Popgen, University of Kiel) genotyped for 587,326 markers on the Affymetrix Axiom Genome-Wide CEU 1 Array. The third data set comprised 856 cases (University Medical Center Groningen) and 2,836 controls (LifeLines) genotyped for 234,758 markers on the Illumina HumanCytoSNP-12. This data set was the discovery data set from Dolmans et al. [18], the first described GWAS for DD. The other two data sets were partly used for replication of 34 discovery SNPs [18] but not analysed for genome-wide association before. We performed sample imputation and phenotypic association testing separately for each of the three platforms. Subsequently, the data from the three GWAS were combined in a genome-wide meta-analysis. See also S1 Fig for an overview of the study design.

### Quality control

The same quality control parameters were applied separately to each data set. Following Dolmans et al. [18], we only considered bi-allelic non-ambiguous SNPs on autosomes and excluded those with a marker call rate <95%, a minor allele frequency <1% or evidence for deviations from Hardy Weinberg equilibrium in controls (p < 10^−4^). Moreover, we removed samples with a call rate <99%, excess homo- or heterozygosity, an ambiguous sex assignment or indication of being an outlier with respect to the genetic composition of the other samples. In addition, for pairs of duplicates or close relatives (average allele sharing identical-by-descent >0.4), the sample with the lower call rate was excluded. The final data sets for analysis comprised 619 (180 cases, 439 controls), 1,530 (441, 1,089) and 3,654 (852, 2,802) samples, respectively.

### Pre-Phasing and imputation

We used SHAPEIT v.2.r790 [23] (https://mathgen.stats.ox.ac.uk/genetics_software/shapeit/shapeit.html#download) for phasing genotype data prior to imputation. Genotype imputation was done with IMPUTE2 v2.3.1 [24] (https://mathgen.stats.ox.ac.uk/impute/impute_v2.html#download). We used reference data from the 1000 Genomes Project [25] (release of June 2014; https://mathgen.stats.ox.ac.uk/impute/data_download_1000G_phase1_integrated_SHAPEIT2_16-06-14.html) for both phasing and imputation. Post-imputation quality control required SNPs to have a minor allele frequency of at least 5% and an information score in the imputation of at least 30%.

### Genome-wide association testing

We used SNPTEST v2.5 [26] (https://mathgen.stats.ox.ac.uk/genetics_software/snptest/snptest.html) for phenotypic association testing under an additive genetic model, using the predicted allele dosage from the imputation. Top-ranking components obtained from a multi-dimensional scaling (MDS) analysis based on genome-wide data of allele sharing identical-by-state were included as fixed effects in the regression-based association test in order to adjust for population stratification. The number of MDS components was determined by minimizing genomic inflation factor (see S1 Table).

### Meta-analysis

We performed the genome-wide meta-analysis using GWAMA v2.1 [27] (http://www.well.ox.ac.uk/gwama/index.shtml). The analysis was restricted to bi-allelic markers that had been studied for an association with DD in at least two cohorts (n = 5,894,478 SNPs). Effect sizes were synthesized using a fixed-effects regression model, thereby hypothesizing that the studies differed only in their statistical power to detect the outcome of interest. Both Cochran’s *Q* statistic and *I*^*2*^ index were used to evaluate statistical heterogeneity between studies [28].

Only SNPs that passed imputation post-hoc quality control and that were studied for an association with Dupuytren’s disease in at least two cohorts were considered in the meta-analysis (n = 5,894,478). SNPs for which Cochran’s *Q* test (p < 0.05) or the *I²* index (*I²* > 25) indicated heterogeneity between studies were excluded (n = 1,543,737). In particular, the global Manhattan plot and QQ plot, both generated via R-script provided by GWAMA, were finally based upon 4,350,741 SNPs. 4,199,404 associations were studied in all three cohorts (96.52%).

### Gene and pathway analysis

#### Search for overrepresented GO terms using Alligator

ALLIGATOR [29] (http://x004.psycm.uwcm.ac.uk/~peter) was used to test for Gene Ontology (GO) categories that were overrepresented in a list of significantly associated SNPs from the meta-analysis. More specifically, out of 4,350,741 SNPs that indicated no heterogeneity between studies, those 185,986 SNPs that were nominally significant (p<0.05) and showed the same effect direction in all three studies were considered for the pathway enrichment analysis. Alligator assigns SNPs to genes based on genomic location. More specifically, a SNP is assigned to a gene if it falls within the gene plus 20kb upstream or downstream of the first and last exon.

#### Gene-based test using VEGAS2

The offline version of VEGAS2 [30] () was used to obtain gene-based p-values from single-SNP p-values. Based on SNP association p-values the software calculates empirical gene-based p-values by a simulation procedure. We based our analysis on those 4,350,741 SNPs that did not show indication of heterogeneity between studies. Estimation of inter-marker linkage disequilibrium was based on the EUR population from the 1000 Genomes Project phase I. Gene boundaries were set to ±50 kb of each gene. Up to 10^6^ simulations were performed per gene. 24,596 genes were tested and genes with p < 2.03x10^-6^ (Bonferroni correction for multiple testing, i. e. 0.05/24,596) were considered to be significantly associated with DD.

#### Connecting genes from associated loci

We used three different software tools to search for relationships between genes from those genetic loci that harbored at least one index SNP with p < 10^−5^ in our meta-analysis:

GRAIL [31] (https://www.broadinstitute.org/mpg/grail/) looks for co-citations of genes from the associated regions in scientific publications. Since GRAIL uses the hg18 genome assembly and CEU HapMap SNPs, genomic regions instead of SNPs were used as query input because most top-ranking SNPs from the meta-analysis were not present in HapMap. Regions +/-50kb around the top-ranking SNP of each region were used as query regions. Chromosomal positions were translated to hg18 coordinates with the UCSC LiftOver tool (http://genome.ucsc.edu/cgi-bin/hgLiftOver) and used as query/seed regions for GRAIL analysis.

DAPPLE2 [32] (http://www.broadinstitute.org/mpg/dapple/dappleTMP.php) searches for connections between query genes in a high confidence protein-protein interaction (PPI) dataset (InWeb). We used SNPs, genomic regions, and genes as queries. Direct and indirect connections are given by the software and parameters calculated by random permutation to access the significance of the found connections.

In PrixFixe [33] (http://llama.mshri.on.ca/~mtasan/GranPrixFixe/html/) SNPs reported by GWAS act as seeds for LD-based query regions. We assessed connections between genes based on shared function using PrixFixe. Functional similarities between genes across distinct candidate sets were then located and genes scored based on the frequency and strength of identifying such functional connections.

#### Network-based pathway analysis

We performed a network-assisted search for enriched protein-protein interactions (PPIs) based on all gene-based p-values from the VEGAS2 analysis. We used two different software tools for this approach, namely dmGWAS in R and Cytoscape (see below). For both tools, we used the 2014 version of the Reactome FI (http://www.reactome.org/) as PPI reference dataset. This dataset contains 11,879 nodes and 217,249 edges and is by design enriched for true biologically functional relationships [34].

The R package dmGWAS 3.0 [35] (http://bioinfo.mc.vanderbilt.edu/dmGWAS) was used to search for enriched modules in Dupuytren´s disease using gene-based p-values as node weights and differential co-expression of genes as edge weights (based on the whole transcriptome data set). The EW_dmGWAS algorithm implemented in dmGWAS 3.0 integrates GWAS signals and gene expression profiles to extract dense modules from the background PPI network. Node weights are derived from GWAS gene-based p-values and edge weights are derived from gene expression profiling. The resulting module score is a combination of node weight and edge weight.

PINBPA [36] Cytoscape (http://www.cytoscape.org/) also searches for PPI in GWAS datasets. Genes with a p-value < 0.05, 0.1 and 0.2 were used as seed genes for a greedy search algorithm-based search for connected modules. Genes from the gene-based test (VEGAS2) were mapped to the Reactome FI network. All mapped genes with a p-value < 0.05 (or 0.1 and 0.2, respectively) were used as seed genes in the greedy search for enriched modules. We considered modules with a z-score > 5 and a network-size ≥ 5.

### Transcriptome analysis

The Illumina HumanHT-12 v3 Expression BeadChip was used for whole genome expression analysis of disease samples and controls. Twelve primary tissue samples from patients were compared with 12 sex and age matched controls. Total RNA was extracted from 150mg RNAlater stabilised tissue by phenol/chloroform extraction. The tissue was cut into very small pieces and homogenised in a beadmill (Tissue Lyser 2, Qiagen, Germany) for 2–6 min at 30s^-1^ and room temperature. The phenol/chloroform purified RNA was precipitated with ethanol; dried and resuspended in RNase free water. RNA concentration and integrity were assessed with a 2100 Bioanalyzer (Agilent, Germany). For the whole-genome gene expression analysis only RNA samples with RNA integrity number (RIN) ≥ 6.9 were used. The Illumina TotalPrep RNA Amplification Kit (Ambion, USA) was applied for generating biotinylated, amplified RNA for hybridisation and samples were hybridised with the Illumina HumanHT-12 v3 Expression BeadChip according to the manufactures’ protocol. Data were analysed with the Illumina GenomeStudio Data Analysis Software and normalised, the background was subtracted, the Illumina custom error model applied and p-values were adjusted for multiple testing (Benjamini-Hochberg correction). Ingenuity Pathways Analysis (IPA) software was used to identify altered pathways and protein networks in DD. Microarray data were submitted to the Gene Expression Omnibus (GEO) database (accession number: GSE75152).

## Results

### GWAS meta-analysis in DD

**Fig 1:**
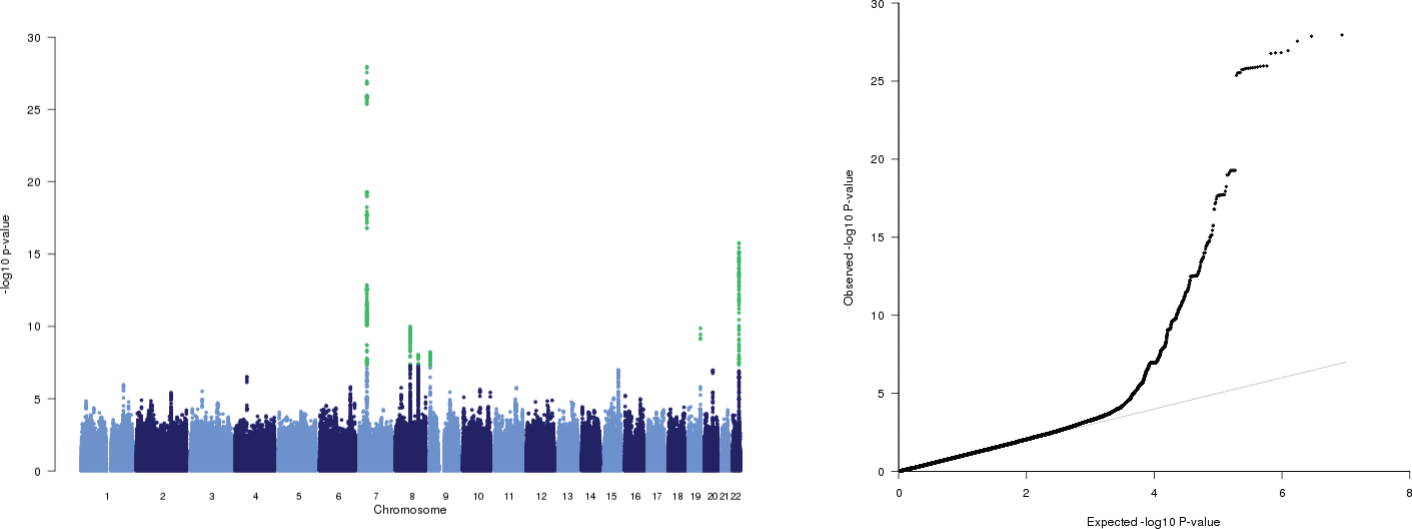
Results of the Genome-Wide Meta-Analysis in Dupuytren’s Disease. P-values were obtained under an additive genetic model with adjustment for minor population stratification. Effect sizes were synthesized using a fixed-effects regression model, thereby hypothesizing that the studies differed only in their statistical power to detect the outcome of interest. Both Cochran’s *Q* statistic and *I*^*2*^ index were used to evaluate statistical heterogeneity between studies. (A) Quantile-quantile (QQ) plot of observed phenotypic association p-values. The grey line represents concurrence of the expected and the observed p-values under a true null hypothesis of no association. Values above the line indicate a signal in the data. (B) Manhattan plot showing the p-values (−log_10_) plotted against their respective positions on each chromosome with each dot representing a SNP. Green dots represent SNPs that surpass the genome-wide significance threshold of 5×10^−8^. Manhattan and QQ plots were generated based on those 4,350,741 SNPs included in the meta-analysis that did not show indication of heterogeneity between studies.

Here we performed genome-wide genotype imputation and meta-analysis for three GWAS data sets for DD, which comprise 5,803 samples after quality control, from Germany, Switzerland, and the Netherlands (S1 Fig). Manhattan and QQ plots were generated based on those 4,350,741 SNPs from the meta-analysis that did not indicate heterogeneity between studies (Fig 1). In the subsequent analysis only SNPs that showed the same direction of effect in all three data sets were considered (n = 4,199,404 SNPs) (S2 Table). Overall, the meta-analysis yielded 910 SNPs showing suggestive association with DD (p <10^−5^), located in 23 chromosomal regions. Out of these, 371 SNPs reached genome-wide significance (p <5×10^−8^). These SNPs are located on chromosomes 7p14.1 (n = 162), 8q13.2 (n = 79), 8q23.1 (n = 29), 9p24.3 (n = 25), 19q13.43 (n = 6), and 22q13.31 (n = 70). The top-ranking SNP from each of these loci is given in Table 1. We confirmed six of the nine loci previously shown to be associated with DD [18] on a genome-wide significant level. As in Dolmans et al. [18], we observed the strongest association signal on chromosome 7p14.1. Here the imputed SNP rs17171229 (p = 1.11x10^-28^, OR 2.02) gave a slightly stronger signal in the meta-analysis than the previous GWAS top SNP, rs16879765 (p = 1.52x10^-27^, OR 2.02). SNPs rs17171229 and rs16879765 are in close linkage disequilibrium (r^2^ = 0.93; based on 1000 Genomes Project EUR phase 1 population). For one locus that previously showed genome-wide significance, on chromosome 20q12, the top SNP in our meta-analysis, rs3577 located in the 5’ UTR of *MAFB* reached a p-value of 1.12x10^-7^. We did not observe suggestive association signals for two previously genome-wide significant loci, on chromosomes 1p36.12 (including *WNT4*) and 7q31.2 (*WNT2*). On chromosome 1p36.12, rs7524102 associated with DD in Dolmans et al. [18], showed a p-value of 4.88x10^-5^ (OR 1.28). The SNP with the lowest p-value in this region was rs7537281 (p = 1.48x10^-5^; OR 1.30). SNP rs4730775 (within *WNT2*) on chromosome 7q31.2 showed a p-value of 0.000791 (OR 0.85). The SNP with the lowest p-value in this region was rs56145820 (p = 5.81x10^-5^; OR 1.28).

**Table 1:** Top Ranking SNPs of the Genome-Wide Meta-Analysis in Dupuytren’s Disease.

SNP	Chr	Position	A1/A2	OR	p-value	Direction of effect	Gene(s)	Reference
rs896319	1	205205651	G/T	0.71	1.08x10^-6^	—	DSTYK, CNTN2	this report
rs12612573	2	167345393	C/G	1.26	3.86x10^-6^	+++	SCN9A	this report
rs12493910	3	55909752	A/T	1.31	3.14x10^-6^	+++	ERC2, WNT5A	this report
rs59885791	4	55943950	G/A	0.67	3.06x10^-7^	—	KDR	this report
rs11155615	6	149361706	A/G	1.28	1.57x10^-6^	+++	TAB2, UST, SUMO4	Dolmans^a^
rs17171229	7	37996682	T/C	2.02	**1.11x10**^**-28**^	+++	SFRP4, EPDR1	Dolmans^a^
rs1485748	8	25870252	A/G	1.27	1.76x10^-6^	+++	DPYSL2, EBF2	this report
rs13279808	8	69992479	G/T	1.36	**1.02x10**^**-10**^	+++	SULF1	Dolmans^a^
rs422847	8	109103274	A/G	0.76	**9.29x10**^**-9**^	—	EIF3E, RSPO2	Dolmans^a^
rs183228744	8	145501808	A/G	0.77	7.85x10^-6^	—	BOP1	this report
rs7867335	9	1200400	T/C	1.39	**6.24x10**^**-9**^	+++	DMRT2	Dolmans^a^
rs28436028	9	97426576	A/G	1.43	3.58x10^-6^	+++	C9orf3, FBP1	this report
rs138852017	10	4068310	G/A	1.51	8.04x10^-6^	+++	KLF6	this report
rs10786118	10	82663861	T/C	0.80	2.35x10^-6^	—	TSPAN14, FAM213A, SH2D4B	this report
rs61862987	10	132907934	A/G	1.39	3.71x10^-6^	+++	TCERG1L	this report
rs661148	11	105700326	C/A	1.38	1.78x10^-6^	+++	CARD18, GRIA4, U4	this report
rs12899214	15	67302803	G/A	1.23	9.02x10^-6^	+++	SMAD3	this report
rs4932195	15	89252781	T/C	0.73	1.02x10^-7^	—	ACAN, ISG20	Dolmans^a^
rs7189020	16	304803	A/T	1.25	7.03x10^-6^	+++	AXIN1, STUB1	this report
rs184572725	16	1305097	G/T	1.55	6.26x10^-6^	+++	TPSD1	this report
rs60333289	19	57677926	G/T	1.43	**1.37x10**^**-10**^	+++	AURKC, DUXA	Dolmans^a^
rs3577	20	39314670	G/A	0.68	1.12x10^-7^	—	PLCG1, MAFB	Dolmans^a^
rs28502139	22	46396538	C/T	1.65	**1.75x10**^**-16**^	+++	WNT7B	Dolmans^a^

p-values that surpass the level of genome-wide significance are depicted in bold.

A1 –reference allele; A2 –alternative allele; OR–odds ratio

^a^ Dolmans et al. [18]

Of the remaining 16 regions that contained SNPs with p-values <10^−5^ two have been previously identified: one on chromosome 6q25.1 near *TAB2* (rs11155615, top-ranking SNP from the meta-analysis, located intronic of *UST*), and the other one on chromosome 15q26.1 with the top-ranking SNP from the meta-analysis, rs4932195, located between the genes *ISG20* and *ACAN*. Moreover, we identified 14 new suggestive loci on chromosomes 1q32.1, 2q24.3, 3p14.3, 4q12, 8p21.2, 9q22.32, 10p15.1, 10q23.1, 10q26.3, 11q22.3, 15q22.31-q23, 15q25.3-q26 and 16p13.3 (2 hits) with p<10^−5^ (Table 1). These loci require replication in an independent sample set.

### Search for connections between potentially associated loci

In total, we identified 23 loci with suggestive evidence for association with DD. All these were used for pathway analysis with GRAIL, DAPPLE2 and PrixFixe to identify the most likely candidate gene(s) from each region based on connectivity. The candidate gene(s) with the best scores/lowest p-values for each region are given in Table 2. In order to allow comparison, Table 2 also contains the gene(s) with the lowest p-values for each region from the VEGAS2 analysis. All three software tools unanimously identified *SFRP4*, *KDR* and *WNT7B* as the best candidate genes in their respective loci. *SFRP4* and *WNT7B* locate to the two regions with the lowest p-values in our study. For one region, on 10q23.1 (rs10786118), no candidate genes were identified by any software tool. Most interestingly, additional Wnt signalling components were identified in regions with suggestive p-values, among them *WNT5A* on chromosome 5p14.3 and *AXIN1* on chromosome 16p13.3. *AXIN1* locates to a gene rich region with more than thirty potential candidate genes.

**Table 2:** Genes Identified by Pathway Analysis for the Top 23 Meta-Analysis Loci.

Chr	SNP	Meta p-value	GRAIL gene	GRAIL p-pvalue	DAPPLE2 gene	DAPPLE2 p-value	PrixFixe gene	PrixFixe GA score	VEGAS2 gene	VEGAS2 p-value
1	rs896319	1.08x10^-6^	RIPK5, TMCC2	1	NUAK2	0.072	CNTN2	0.48	DSTYK	1.31x10^-4^
2	rs12612573	3.86x10^-6^	SCN7A	0.184	NA	NA	SCN7A	0.14	SCN7A	6.30x10^-5^
3	rs12493910	3.14x10^-6^	ERC2	0.604	ERC2	NA	WNT5A	0.62	ERC2	0.016
4	rs59885791	3.06x10^-7^	KDR	0.307	KDR	0.488	KDR	0.47	KDR	0.178
6	rs11155615	1.57x10^-6^	UST	0.588	NA	NA	TAB2	0.35	UST	0.005
7	rs17171229	**1.11x10**^**-28**^	SFRP4	0.001	SFRP4	0.236	SFRP4	0.59	SFRP4, EPDR1, NME8	1.00x10^-6^
8	rs1485748	1.76x10^-6^	EBF2	0.285	NA	NA	BNIP3L	0.21	EBF2	0.005
8	rs13279808	**1.02x10**^**-10**^	NA	NA	NA	NA	SULF1	0.36	LOC100505718	1.00x10^-6^
8	rs422847	**9.29x10**^**-9**^	RSPO2	0.001	NA	NA	EIF3E	0.22	EIF3E	2.00x10^-6^
8	rs183228744	7.85x10^-6^	SCXB	0.014	FBXL6	0.311	SCRIB	0.47	SCXB	1.02x10^-4^
9	rs7867335	**6.24x10**^**-9**^	NA	NA	NA	NA	NA	NA	DMRT2	0.569
9	rs28436028	3.58x10^-6^	FBP1	0.531	FBP2	0.429	FBP2	0.19	FBP1	0.002
10	rs138852017	8.04x10^-6^	NA	NA	NA	NA	KLF6	0.16	MIR6078	0.019
10	rs10786118	2.35x10^-6^	NA	NA	NA	NA	NA	NA	DYDC2	0.007
10	rs61862987	3.71x10^-6^	TCERG1L	0.718	NA	NA	NA	NA	TCERG1L	0.477
11	rs661148	1.78x10^-6^	GRIA4	1	GRIA4	0.260	AASDHPPT	0.12	GRIA4	2.44x10^-4^
15	rs12899214	9.02x10^-6^	NA	NA	SMAD3	0.522	SMAD3	0.46	SMAD3	0.041
15	rs4932195	1.02x10^-7^	NA	NA	ACAN	NA	ACAN	0.40	ISG20	1.00x10^-6^
16	rs7189020	7.03x10^-6^	AXIN1	0.007	PDIA2	0.082	AXIN1	0.54	LUC7L	1.60x10^-6^
16	rs184572725	6.26x10^-6^	CACNA1H	0.559	TPSAB1	0.135	NA	NA	TPSD1	0.052
19	rs60333289	**1.37x10**^**-10**^	ZNF264	0.434	AURKC	NA	ZIM2	0.31	ZNF264	2.00x10^-6^
20	rs3577	1.12x10^-7^	MAFB	0.006	MAFB	0.591	PLCG1	0.53	MAFB	3.16x10^-4^
22	rs28502139	**1.75x10**^**-16**^	WNT7B	0.001	WNT7B	0.095	WNT7B	0.60	WNT7B, LOC730668, LINC00899, PRR34, LOC150381, MIRLET7BHG, MIR3619	1.00x10^-6^

Candidate genes identified by three different pathway tools, GRAIL, DAPPLE2 and PrixFixe, and the gene-based test with VEGAior the 23 meta-analysis loci.

NA–no data available (no candidate gene found).

GRAIL identified one or more candidate genes for 17 out of 23 regions, with four genes, *SFRP4*, *WNT7B*, *AXIN1* and *RSPO2*, having corrected p-values <0.01. When using candidate SNPs as input, DAPPLE2 identified genes in 10 out of 23 regions. Connectivity among these genes was not higher than expected by chance (and no single gene with p-values < 0.05). When regions +/-50kb around the top-ranking SNP were used as input/seeds in the DAPPLE2 analysis (similar to GRAIL input), DAPPLE2 considered 13 out of 23 regions in the analysis and identified two direct connections, between *WNT7B* and *SFRP4* and between *SMAD3* and *AXIN1*. Again, the identified genes were not more connected than expected by chance (i. e. no significant p-values for overall connectivity and for single candidate genes). PrixFixe identified candidate genes in 19 regions. The software does not give p-values but a normalised scores between 0 and 1, where a higher score indicates higher connectivity. Three genes, *WNT5A*, *SULF1* and *TAB2*, were uniquely identified by PrixFixe.

Functional annotation for all 13 non-redundant genes with p-values <0.01 or scores >0.4 with DAVID [37] resulted in the best enrichment cluster (enrichment score 4.1) for Wnt signalling (GO term GO:0016055, p-value 0.002 (Bonferroni corrected), KEGG pathway hsa04310, p-value 7.56x10^-4^, SP-PIR keyword “wnt signaling pathway”, p-value 4.76x10^-5^) and included five genes, *WNT5A*, *WNT7B*, *SFRP4*, *RSPO2* and *AXIN1*.

### Search for connections between GWAS loci

While we first restricted our search for functionally connected genes to the 23 regions showing at least suggestive association in our meta-analysis, we subsequently used the software tool Alligator to identify overrepresented GO terms in the whole meta-analysis data set. For 141 out of 2,631 analysed GO categories, the category-specific p-value for overrepresentation was <0.05 when using a cutoff of p<0.01 for defining significant SNPs (S4 Table). The number of all functional types of GO categories (i.e. cellular components, molecular functions, and biological processes) that reached significance levels for overrepresentation of 0.05, 0.01, and 0.001 are shown in S5 Table.

Significance of the number of overrepresented categories was observed when using less stringent p-values for defining significant SNPs (i.e. p<0.05 or p<0.01). This suggests that the genetic susceptibility to DD may involve risk alleles with rather small individual effects. In addition, excess of overrepresented categories was most significant when using 0.01 as the significance criterion defining overrepresentation. Associations for DD are thus supposed to be concentrated in a moderate number of categories, with each of them showing strong evidence of overrepresentation. The three highest ranking overrepresented pathways were “positive regulation of cellular protein metabolic process” (GO:0032270, p-value 0), “positive regulation of protein metabolic process” (GO:0051247, p-value 0) and “response to drug” (GO:0042493, p-value 2x10^-4^).

### Gene-based analysis

We used VEGAS2 to obtain gene-based p-values for phenotypic association from SNP-based p-values, using the 1000 Genomes Project EUR phase 1 reference set. The gene with the lowest p-value for each of the 23 candidate loci is given in Table 2. Overall, the gene-based test mirrors the SNP-based GWAS results. For 14 genes the p-values exceeded a Bonferroni-corrected threshold of p<2.03x10^-6^, including *SFRP4*, *EPDR1* and *NME8* on chromosome 7p14.1–14.2, *LINC01592* on chromosome 8q13.2, *EIF3E* on chromosome 8q23.1, *ISG20* on 15q25.3–26.1, *ZNF246* on 19q13.43 and *WNT7B*, *LOC730668*, *LINC00899*, *PRR34*, *PRR34-AS1*, *MIRLET7BHG*, and *MIR3619* on 22q13.31. Among these genes *ISG20* is the only gene without adjacent genome-wide significant SNPs. However, a SNP near *ISG20*, rs4932194, showed suggestive association with DD in Dolmans et al [18].

### Differential gene expression and pathway analysis

Next we compared transcriptome levels in 12 primary disease tissue samples with that of 12 control tissue samples (S1 Fig). Cases and controls clearly clustered into two distinct groups based on whole transcriptome expression patterns. We considered only transcripts with a detection p-value of 0 in either one or both sample sets. 782 transcripts were upregulated with a fold change ≥2 (S6 Table) and 865 transcripts were downregulated with a fold change of ≤-2 (S7 Table). Among the most upregulated transcripts with fold changes >10 (N = 75) were extracellular matrix components including *THBS4*, *TCN*, *COL11A1*, *COL13A1*, *POSTN*, but also other relevant genes such as *TGFB2*, *ITGA10* and *WISP1*. Among the most downregulated transcripts with a fold change <10 (N = 80) were genes related to muscle function/contraction, notably *ACTA1* and others. Considering the importance of Wnt signalling for the development of DD, we specifically searched for differential expression of the genes involved. From all Wnt genes covered by the array, however, only *WNT2*, *WNT5B* and *WNT11* passed our quality criteria. *WNT2* showed no differential expression, *WNT5B* was 2.6-fold upregulated and *WNT11* was 3.5-fold downregulated in DD cases.

Genes with a fold change cutoff of ≥1.4 were further investigated in the IPA core analysis (N = 4,062; S8–S12 Tables). The top canonical pathway was the Wnt/β-catenin signalling pathway (ratio 60/166 (0.361), p-value 1.57x10^-6^, z-score 0.302). A slightly positive z-score indicates upregulation of this pathway in DD. The second highest ranking pathway was “LPS/IL-1 Mediated Inhibition of RXR Function” (ratio 70/208 (0.337), p-value 4.40x10^-6^, z-score 1.80) and the third highest ranking pathway was “Hepatic Fibrosis/Hepatic Stellate Cell Activation” (ratio 66/196 (0.337), p-value 7.97x10^-6^, z-score NA). The top network in the IPA analysis contained 68 nodes and a score of 42, with the seed gene *CTNNB1* encoding β-catenin. Functional annotation of those 68 genes with DAVID and Enrichr [38] revealed Wnt signalling as the best enriched signalling pathway for these genes. The top upstream regulator identified in the IPA core analysis was *TGFB1*, predicted to be activated with a p-value of 4.96x10^-13^ and an activation z-score of 3.75. *TGFB1* had 110 target molecules in the analyzed data set. The top biological function predicted to be activated with a z-score of 2.89 and a p-value of 4.81x10^-25^ was “proliferation of cells”.

### Network-based pathway analysis

We further used two software tools for whole-genome pathway analysis, dmGWAS and the cytoscape app iPINBPA. Both tools apply a greedy search algorithm to search for enriched modules.

For dmGWAS we considered 24,596 genes with p-values and 3,873 genes with expression values (fold change cutoff 1.4). The final background network had 2,165 nodes and 7,737 edges. The default lambda calculated by the software was 0.47. The analysis resulted in 943 modules with normalised module scores (Sn) between 18.91 and 0.76. We selected the top 5% modules for further analysis to have sufficient numbers of genes for functional annotation clustering. Within these 121 genes there were two genes from genomic regions with SNP-based p-values <10^−5^, *MAFB* and *ISG20*. A full list of the genes is given in S7 Table. Functional annotation for the 121 genes with DAVID [37] yielded 79 enrichment clusters. The best cluster had an enrichment score of 6.76 and consisted of sixteen GO terms concerning fiber contraction and the (actin) cytoskeleton. Other clusters concerned functions related to contraction, integrin, adhesion, and the ECM (S13 and S14 Tables).

In contrast to dmGWAS, iPINBPA can restrict the overlap between modules resulting in bigger top modules with a default of 20% maximal overlap. The top module (S15 Table) from the iPINBPA analysis contained 851 nodes and had a z-score of 44.9. The start gene for this module was *MYC*. The mean gene-based p-value from the top modules was 0.12±0.08 and the mean SNP-based p-value was 0.01±0.02. Five genes from the top GWAS meta-analysis regions were included in this network, namely *AXIN1*, *EIF3E*, *ISG20*, *MAFB*, *SFRP4*, and *RBBP5*.

Functional annotation of the 851 genes with DAVID [37] resulted in 320 enrichment clusters (S16 Table). The best enrichment cluster had a score of 26.77 and consisted of six cellular component GO terms, all concerning nuclear lumen and nucleoplasm. Interestingly the 14^th^ best enrichment cluster, with a score of 5.74, concerned Wnt signalling with the following 29 genes: *NKD2*, *WNT5B*, *CAMK2G*, *PPP2R5C*, *TCF7L2*, *CTNNB1*, *WNT2*, *RAC1*, *PPP3CC*, *FRAT2*, *PLCB1*, *AXIN2*, *MYC*, *FBXW11*, *AXIN1*, *DVL2*, *TBL1XR1*, *DVL3*, *ROCK2*, *CREBBP*, *SMAD3*, *FZD7*, *CTNNBIP1*, *SENP2*, *CCND1*, *PRICKLE1*, *GSK3B*, *SFRP4*, *PPP2R5E*. We also analysed the list of 851 genes with the Enrichr [38] tool (S17 Table). Among the top ten pathways associated with the iPINBPA top module were Wnt signalling, Notch signalling and focal adhesions. The overlap between the top 5% from dmGWAS and top module from iPINBPA were 40 genes (p-value of overlap <4.2x10^-17^ based on 11,879 genes in the Reactome PPI data). These 40 genes are shown in S11 Table. Among these genes were *AXIN2*, *IGF1*, *ISG20*, *ITGB1*, *LAMB1*, *MAFB* and others. The mean gene-based p-value for these 40 genes was 0.086±0.073 and the mean SNP-based p-value was 0.005±0.006. These values are thus slightly smaller than the mean p-values for the individual gene lists from dmGWAS and iPINBPA.

## Discussion

We performed a genome-wide meta-analysis as well as enrichment and functional analysis for Dupuytren’s disease (DD), based on three data sets comprising in total 1,580 cases and 4,480 control samples from Germany, Switzerland and the Netherlands. We only considered those SNPs that showed the same direction of effect in all three data sets in order to increase the robustness of our results, restricting the analysis to 4,199,404 SNPs in total. Because of the still relatively small sample size, we opted for including all samples in one meta-analysis and did not split the samples into discovery and replication data sets. A weakness of this study is, thus, the current lack of replication in an independent data set. Nevertheless, our approach of a genome-wide meta-analysis after stringent quality procedure application in all data sets is likely to have revealed robust findings of suggestive evidence for new susceptibility loci and important insight into disease mechanisms involved in the pathogenesis of DD.

First, we have confirmed six of the nine previously described loci associated with DD on genome-wide significance level in an enlarged data set. Second, we have identified 14 new suggestive loci, with p-values ranging between 1x10^-7^ and 9x10^-6^. In any case, these loci require confirmation and replication in an independent data set. Intriguingly, however, several new loci of suggestive evidence again contain genes related to Wnt signalling. These genes include *WNT5A* on chromosome 3p14.3, which is a ligand of frizzled receptors; *EBF2* on 8p21.2, which acts in synergy with the Wnt-responsive LEF-1/β-catenin pathway; *SMAD3* on 15q22.33, which is involved in positive regulation of the canonical Wnt signalling pathway; and *AXIN1* on 16p13.3, which is a component of the β-catenin destruction complex. These observations clearly support the notion that the suggestive loci identified in this study may be indeed true signals, they corroborate the outstanding importance of Wnt signalling in the susceptibility for DD and meet the expectation that Wnt signalling pathway related genes were also located in genomic regions with weaker association signals. Our results provide evidence in addition to previous observations that Wnt signalling is involved in the genetic basis of the disease.

The observed distribution of small p-values derivates early from the expected distribution as seen in the QQ plot, both for the SNP-based and gene-based data. Even after stringent correction for genomic inflation (population stratification) a large number of SNPs (and genes) with small p-values remain. Although data from a larger sample set strictly corrected for population stratification are necessary to substantiate this observation regarding Dupuytren’s disease, a broad genetic basis is common in truly complex genetic diseases [39, 40]. About 6% of all tested genes had p-values <0.05 in the gene-based association test conducted with VEGAS2. This is similar to the percentages reported recently, for instance, for three schizophrenia GWAS data sets described by Chang et al [41]. Assuming many loci involved in the susceptibility for the disease, genome-wide network analysis may help to identify true genetic associations with small effect sizes in the increasing noise of false positives with larger p-values. Predicting the importance of a functional module is often easier than that of single genes [42].

In this study, we have performed a comprehensive pathway and network analysis for DD using data from GWAS meta-analysis mainly with two different software tools. Both tools use a greedy search algorithm to search for densely connected modules that are enriched for small p-values. We increased the power to detect functionally relevant PPI networks by integrating GWAS with differential gene expression data. Indeed, our results indicate a broad functional spectrum for the genetic basis of DD, covering many small effect size variants supposed to play a role. It is apparent from our analysis that in addition to the Wnt signalling, other signalling pathways and biological processes, notably focal adhesions and Notch signalling, may play a role. This is in concordance with the observations that complex diseases, with the probable exception of immune diseases, may be genetically based on a number of different (signalling) pathways [43, 44]. The Wnt and Notch signalling pathways are both highly conserved, and heavy crosstalk is known to take place between them during development and disease [45]. Knowledge of the interplay of different pathways will ultimately help to understand pathogenic mechanisms and design treatment options for a disease [46]. Notably, functional annotation of gene lists is only the first step to functional characterization especially in the context of disease and affected tissues.

In our network analysis many factors point to the importance of ECM and focal adhesion, especially the results from dmGWAS, where the network analysis was based on GWAS and gene expression data. The expression of many ECM component genes was found to be upregulated in DD tissues compared to control tissues in our and other studies [47–51]. This network analysis may help to identify those ECM components deregulated in DD that are critically influenced by genetic factors. Of note, some of the genes from the suggestive GWAS regions code for ECM components or secreted proteins, such as *ACAN* and *TPSD1*.

Whole-transcriptome analysis correlated with the GWAS data results in that it highlighted the Wnt signalling pathway to be differentially expressed in disease compared to control tissue. Our transcriptome results support the GWAS findings, highlighting the canonical Wnt/β-catenin pathway to be significantly altered in disease tissues compared to control tissues. Although there are numerous expression studies published in DD [47–51] none of them reported upregulation of Wnt/β-catenin signalling so far, though upregulation and the accumulation of nuclear β-catenin levels in DD tissues have been described [52, 53]. Moreover, micro-RNA profiling in DD showed deregulation of β-catenin in DD recently [54]. Other deregulated pathways concerned immune responses and fibrotic processes, which might fit well to the proposed disease mechanisms. The observed downregulation of *WNT11* in patient tissues is in accordance with previous findings [55].

## Conclusion

We replicated for the first time previously identified susceptibility loci for DD in a meta-analysis of three imputed GWAS data sets. Our findings further corroborate the strong genetic basis and the wide importance of Wnt signalling genes in the susceptibility for DD. In addition, the results demonstrate that genome-wide imputation increases the power of association studies for finding new risk loci for DD. Moreover, we performed whole transcriptome analysis of DD tissues compared with control tissues and found the Wnt signalling pathway significantly deregulated in DD. In summary, our pathway and network analyses, integrating GWAS and transcriptome data, indicate that DD may have a broad genetic basis. The pathogenesis involves Wnt signalling and several other biological processes including the regulation of focal adhesions and the deposition of the ECM.

## Supporting Information

S1 Fig Overview of study design, meta-analysis, and network analysis. (TIF)

S1 Table Summary of the three data sets used for association testing before and after quality control. (XLSX)

S2 Table Summary of association testing. (XLSX)

S3 Table Summary of the samples used in the whole-genome gene expression analysis. (XLSX)

S4 Table Overrepresented GO terms in DD. (XLSX)

S5 Table Number of overrepresented GO terms in DD with different p-value cutoffs. (XLSX)

S6 Table Upregulated transcripts. (XLSX)

S7 Table Downregulated transcripts. (XLSX)

S8 Table IPA top networks. (XLSX)

S9 Table IPA top regulator effects. (XLSX)

S10 Table IPA top upstream analysis. (XLSX)

S11 Table IPA top disease and function. (XLSX)

S12 Table IPA top pathway—Wnt signalling. (XLSX)

S13 Table Genes from dmGWAS top 5% modules. (XLSX)

S14 Table Functional annotation with DAVID for the 121 genes from the dmGWAS top 5% modules. (XLSX)

S15 Table Genes from iPINBPA top subnetwork. (XLSX)

S16 Table DAVID functional enrichment analysis of the top network from iPINBPA. (XLSX)

S17 Table Overlap between dmGWAS 5% and iPINBPA top module. (XLSX)

## References

[1] Early PF. Population studies in Dupuytren’s contracture. J Bone Joint Surg. 1962;44B: 602–13.

[2] Brenner P, Krause-Bergmann A, Van VH. [Dupuytren contracture in North Germany. Epidemiological study of 500 cases]. Unfallchirurg. 2001;104(4): 303–11. Epub 2001/05/19. . 11357696 10.1007/s001130050732

[3] Hindocha S, McGrouther DA, Bayat A. Epidemiological evaluation of Dupuytren's disease incidence and prevalence rates in relation to etiology. Hand (N Y). 2009;4(3): 256–69. Epub 2009/01/16. 10.1007/s11552-008-9160-9 19145463 PMC2724613

[4] Gudmundsson KG, Arngrimsson R, Sigfusson N, Jonsson T. Increased total mortality and cancer mortality in men with Dupuytren's disease: a 15-year follow-up study. J Clin Epidemiol. 2002;55(1): 5–10. Epub 2002/01/10. . 11781116 10.1016/s0895-4356(01)00413-9

[5] Lanting R, van den Heuvel ER, Westerink B, Werker PM. Prevalence of Dupuytren disease in The Netherlands. Plast Reconstr Surg. 2013;132(2): 394–403. Epub 2013/07/31. 10.1097/PRS.0b013e3182958a33 . 23897337

[6] Burge P. Genetics of Dupuytren's disease. Hand Clinics. 1999;15(1): 63–71. Epub 1999/03/02. . 10050243

[7] Burge SK, Amodei N, Elkin B, Catala S, Andrew SR, Lane PA, et al An evaluation of two primary care interventions for alcohol abuse among Mexican-American patients. Addiction. 1997;92(12): 1705–16. Epub 1998/05/15. . 9581003

[8] Godtfredsen NS, Lucht H, Prescott E, Sorensen TI, Gronbaek M. A prospective study linked both alcohol and tobacco to Dupuytren's disease. J Clin Epidemiol. 2004;57(8): 858–63. Epub 2004/10/16. 10.1016/j.jclinepi.2003.11.015 . 15485739

[9] Gudmundsson KG, Arngrimsson R, Sigfusson N, Bjornsson A, Jonsson T. Epidemiology of Dupuytren's disease: clinical, serological, and social assessment. The Reykjavik Study. J Clin Epidemiol. 2000;53(3): 291–6. Epub 2000/04/13. . 10760640 10.1016/s0895-4356(99)00145-6

[10] Liss GM, Stock SR. Can Dupuytren's contracture be work-related?: review of the evidence. Am J Ind Med. 1996;29(5): 521–32. Epub 1996/05/01. 10.1002/(SICI)1097-0274(199605)29:5<521::AID-AJIM12>3.0.CO;2-2 . 8732927

[11] Descatha A, Jauffret P, Chastang JF, Roquelaure Y, Leclerc A. Should we consider Dupuytren's contracture as work-related? A review and meta-analysis of an old debate. BMC Musculoskelet Disord. 2011;12: 96 Epub 2011/05/18. 10.1186/1471-2474-12-96 21575231 PMC3123614

[12] Ling RS. The Genetic Factor in Dupuytren's Disease. J Bone Joint Surg. 1963;45(4): 709–18. Epub 1963/11/01. . 14074318

[13] Coert JH, Nerin JP, Meek MF. Results of partial fasciectomy for Dupuytren disease in 261 consecutive patients. Ann Plast Surg. 2006;57(1): 13–7. Epub 2006/06/27. 10.1097/01.sap.0000205819.53215.52 . 16799301

[14] Hindocha S, John S, Stanley JK, Watson SJ, Bayat A. The heritability of Dupuytren's disease: familial aggregation and its clinical significance. J Hand Surg. 2006;31(2): 204–10. Epub 2006/02/14. 10.1016/j.jhsa.2005.09.018 . 16473680

[15] Becker K, Tinschert S, Lienert A, Bleuler PE, Staub F, Meinel A, et al The importance of genetic susceptibility in Dupuytren's disease. Clin Genet. 2015;87(5): 483–7. Epub 2014/04/23. 10.1111/cge.12410 . 24749973

[16] Dolmans GH, de Bock GH, Werker PM. Dupuytren diathesis and genetic risk. J Hand Surg. 2012;37(10): 2106–11. Epub 2012/10/02. 10.1016/j.jhsa.2012.07.017 . 23021175

[17] Larsen S, Krogsgaard DG, Aagaard Larsen L, Iachina M, Skytthe A, Frederiksen H. Genetic and environmental influences in Dupuytren's disease: a study of 30,330 Danish twin pairs. J Hand Surg. 2015;40(2): 171–6. Epub 2014/05/20. 10.1177/1753193414535720 . 24835475 PMC4810018

[18] Dolmans GH, Werker PM, Hennies HC, Furniss D, Festen EA, Franke L, et al Wnt signaling and Dupuytren's disease. N Engl J Med. 2011;365(4): 307–17. Epub 2011/07/08. 10.1056/NEJMoa1101029 . 21732829

[19] Logan CY, Nusse R. The Wnt signaling pathway in development and disease. Annu Rev Cell Dev Biol. 2004;20: 781–810. Epub 2004/10/12. 10.1146/annurev.cellbio.20.010403.113126 . 15473860

[20] Schaub MA, Boyle AP, Kundaje A, Batzoglou S, Snyder M. Linking disease associations with regulatory information in the human genome. Genome Res. 2012;22(9): 1748–59. Epub 2012/09/08. 10.1101/gr.136127.111 22955986 PMC3431491

[21] Civelek M, Lusis AJ. Systems genetics approaches to understand complex traits. Nat Rev Genet. 2014;15(1): 34–48. Epub 2013/12/04. 10.1038/nrg3575 24296534 PMC3934510

[22] Marbach D, Costello JC, Kuffner R, Vega NM, Prill RJ, Camacho DM, et al Wisdom of crowds for robust gene network inference. Nat Methods. 2012;9(8): 796–804. Epub 2012/07/17. 10.1038/nmeth.2016 22796662 PMC3512113

[23] Delaneau O, Marchini J. Integrating sequence and array data to create an improved 1000 Genomes Project haplotype reference panel. Nat Commun. 2014;5: 3934 Epub 2015/02/06. 10.1038/ncomms4934 25653097 PMC4338501

[24] Howie BN, Donnelly P, Marchini J. A flexible and accurate genotype imputation method for the next generation of genome-wide association studies. PLoS Genet. 2009;5(6): e1000529 Epub 2009/06/23. 10.1371/journal.pgen.1000529 19543373 PMC2689936

[25] Abecasis GR, Auton A, Brooks LD, DePristo MA, Durbin RM, Handsaker RE, et al An integrated map of genetic variation from 1,092 human genomes. Nature. 2012;491(7422): 56–65. Epub 2012/11/07. 10.1038/nature11632 23128226 PMC3498066

[26] Marchini J, Howie B, Myers S, McVean G, Donnelly P. A new multipoint method for genome-wide association studies by imputation of genotypes. Nat Genet. 2007;39(7): 906–13. Epub 2007/06/19. 10.1038/ng2088 . 17572673

[27] Magi R, Morris AP. GWAMA: software for genome-wide association meta-analysis. BMC Bioinformatics. 2010;11: 288 Epub 2010/06/01. 10.1186/1471-2105-11-288 20509871 PMC2893603

[28] Higgins JP, Thompson SG, Deeks JJ, Altman DG. Measuring inconsistency in meta-analyses. BMJ. 2003;327: 557–60. PubMed Central PMCID: PMCPMC192859. 12958120 10.1136/bmj.327.7414.557 PMC192859

[29] Holmans P, Green EK, Pahwa JS, Ferreira MA, Purcell SM, Sklar P, et al Gene ontology analysis of GWA study data sets provides insights into the biology of bipolar disorder. Am J Hum Genet. 2009;85(1): 13–24. Epub 2009/06/23. 10.1016/j.ajhg.2009.05.011 19539887 PMC2706963

[30] Mishra A, Macgregor S. VEGAS2: Software for More Flexible Gene-Based Testing. Twin Res Hum Genet. 2014: 1–6. Epub 2014/12/19. 10.1017/thg.2014.79 . 25518859

[31] Raychaudhuri S, Plenge RM, Rossin EJ, Ng AC, Purcell SM, Sklar P, et al Identifying relationships among genomic disease regions: predicting genes at pathogenic SNP associations and rare deletions. PLoS Genet. 2009;5(6): e1000534 Epub 2009/06/27. 10.1371/journal.pgen.1000534 19557189 PMC2694358

[32] Rossin EJ, Lage K, Raychaudhuri S, Xavier RJ, Tatar D, Benita Y, et al Proteins encoded in genomic regions associated with immune-mediated disease physically interact and suggest underlying biology. PLoS Genet. 2011;7(1): e1001273 Epub 2011/01/21. 10.1371/journal.pgen.1001273 21249183 PMC3020935

[33] Tasan M, Musso G, Hao T, Vidal M, MacRae CA, Roth FP. Selecting causal genes from genome-wide association studies via functionally coherent subnetworks. Nat Methods. 2015;12(2): 154–9. Epub 2014/12/23. 10.1038/nmeth.3215 . 25532137 PMC4480866

[34] Wu G, Feng X, Stein L. A human functional protein interaction network and its application to cancer data analysis. Genome Biol. 2010;11(5): R53 Epub 2010/05/21. 10.1186/gb-2010-11-5-r53 20482850 PMC2898064

[35] Wang Q, Yu H, Zhao Z, Jia P. EW_dmGWAS: edge-weighted dense module search for genome-wide association studies and gene expression profiles. Bioinformatics. 2015 Epub 2015/03/26. 10.1093/bioinformatics/btv150 . 25805723 PMC4514922

[36] Network-based multiple sclerosis pathway analysis with GWAS data from 15,000 cases and 30,000 controls. Am J Hum Genet. 2013;92(6): 854–65. Epub 2013/06/05. 10.1016/j.ajhg.2013.04.019 23731539 PMC3958952

[37] Huang da W, Sherman BT, Lempicki RA. Systematic and integrative analysis of large gene lists using DAVID bioinformatics resources. Nat Protoc. 2009;4(1): 44–57. Epub 2009/01/10. 10.1038/nprot.2008.211 . 19131956

[38] Chen EY, Tan CM, Kou Y, Duan Q, Wang Z, Meirelles GV, et al Enrichr: interactive and collaborative HTML5 gene list enrichment analysis tool. BMC Bioinformatics. 2013;14: 128 Epub 2013/04/17. 10.1186/1471-2105-14-128 23586463 PMC3637064

[39] Yang J, Weedon MN, Purcell S, Lettre G, Estrada K, Willer CJ, et al Genomic inflation factors under polygenic inheritance. Eur J Hum Genet. 2011;19(7): 807–12. Epub 2011/03/17. 10.1038/ejhg.2011.39 21407268 PMC3137506

[40] Manolio TA, Collins FS, Cox NJ, Goldstein DB, Hindorff LA, Hunter DJ, et al Finding the missing heritability of complex diseases. Nature. 2009;461(7265): 747–53. Epub 2009/10/09. 10.1038/nature08494 19812666 PMC2831613

[41] Chang S, Fang K, Zhang K, Wang J. Network-Based Analysis of Schizophrenia Genome-Wide Association Data to Detect the Joint Functional Association Signals. PloS One. 2015;10(7): e0133404 Epub 2015/07/21. 10.1371/journal.pone.0133404 . 26193471 PMC4508050

[42] Cho DY KY, Przytycka TM. Chapter 5: Network Biology Approach to Complex Diseases. PLoS Comput Biol. 2012;8(12): e1002820 10.1371/journal.pcbi.1002820 23300411 PMC3531284

[43] Goh KI, Cusick ME, Valle D, Childs B, Vidal M, Barabasi AL. The human disease network. Proc Natl Acad Sci U S A. 2007;104(21): 8685–90. Epub 2007/05/16. 10.1073/pnas.0701361104 17502601 PMC1885563

[44] Oti M, Brunner HG. The modular nature of genetic diseases. Clin Genet. 2007;71(1): 1–11. Epub 2007/01/06. 10.1111/j.1399-0004.2006.00708.x . 17204041

[45] Hayward P, Kalmar T, Arias AM. Wnt/Notch signalling and information processing during development. Development. 2008;135(3): 411–24. Epub 2008/01/15. 10.1242/dev.000505 . 18192283

[46] Collu GM, Hidalgo-Sastre A, Brennan K. Wnt-Notch signalling crosstalk in development and disease. Cell Mol Life Sci. 2014;71(18): 3553–67. Epub 2014/06/20. 10.1007/s00018-014-1644-x . 24942883 PMC11113451

[47] Forrester HB, Temple-Smith P, Ham S, de Kretser D, Southwick G, Sprung CN. Genome-wide analysis using exon arrays demonstrates an important role for expression of extra-cellular matrix, fibrotic control and tissue remodelling genes in Dupuytren's disease. PloS One. 2013;8(3): e59056 Epub 2013/04/05. 10.1371/journal.pone.0059056 23554969 PMC3595223

[48] Zhang AY, Fong KD, Pham H, Nacamuli RP, Longaker MT, Chang J. Gene expression analysis of Dupuytren's disease: the role of TGF-beta2. J Hand Surg. 2008;33(6): 783–90. Epub 2008/08/13. 10.1177/1753193408091352 . 18694919

[49] Satish L, LaFramboise WA, O'Gorman DB, Johnson S, Janto B, Gan BS, et al Identification of differentially expressed genes in fibroblasts derived from patients with Dupuytren's Contracture. BMC Med Genomics. 2008;1: 10 Epub 2008/04/25. 10.1186/1755-8794-1-10 18433489 PMC2377253

[50] Rehman S, Salway F, Stanley JK, Ollier WE, Day P, Bayat A. Molecular phenotypic descriptors of Dupuytren's disease defined using informatics analysis of the transcriptome. J Hand Surg. 2008;33(3): 359–72. Epub 2008/03/18. 10.1016/j.jhsa.2007.11.010 . 18343292

[51] Pan D, Watson HK, Swigart C, Thomson JG, Honig SC, Narayan D. Microarray gene analysis and expression profiles of Dupuytren's contracture. Ann Plast Surg. 2003;50(6): 618–22. Epub 2003/06/05. 10.1097/01.SAP.0000069066.35253.B3 . 12783014

[52] Montgomery E, Lee JH, Abraham SC, Wu TT. Superficial fibromatoses are genetically distinct from deep fibromatoses. Mod Pathol. 2001;14(7): 695–701. Epub 2001/07/17. 10.1038/modpathol.3880374 . 11455002

[53] Varallo VM, Gan BS, Seney S, Ross DC, Roth JH, Richards RS, et al Beta-catenin expression in Dupuytren's disease: potential role for cell-matrix interactions in modulating beta-catenin levels in vivo and in vitro. Oncogene. 2003;22(24): 3680–4. Epub 2003/06/13. 10.1038/sj.onc.1206415 . 12802275

[54] Mosakhani N, Guled M, Lahti L, Borze I, Forsman M, Paakkonen V, et al Unique microRNA profile in Dupuytren's contracture supports deregulation of beta-catenin pathway. Mod Pathol. 2010;23(11): 1544–52. Epub 2010/08/03. 10.1038/modpathol.2010.146 . 20676061

[55] O'Gorman DB, Wu Y, Seney S, Zhu RD, Gan BS. Wnt expression is not correlated with beta-catenin dysregulation in Dupuytren's Disease. J Negat Results Biomed. 2006;5: 13 Epub 2006/09/01. 10.1186/1477-5751-5-13 16942611 PMC1564412

