# Supplementary figures and images for: Meta-Analysis of Genome-Wide Association Studies and Network Analysis-Based Integration with Gene Expression Data Identify New Suggestive Loci and Unravel a Wnt-Centric Network Associated with Dupuytren’s Disease
Source: PLoS One. 2016 Jul 28;11(7):e0158101. doi: 10.1371/journal.pone.0158101 (PMC4965170; doi:10.1371/journal.pone.0158101)

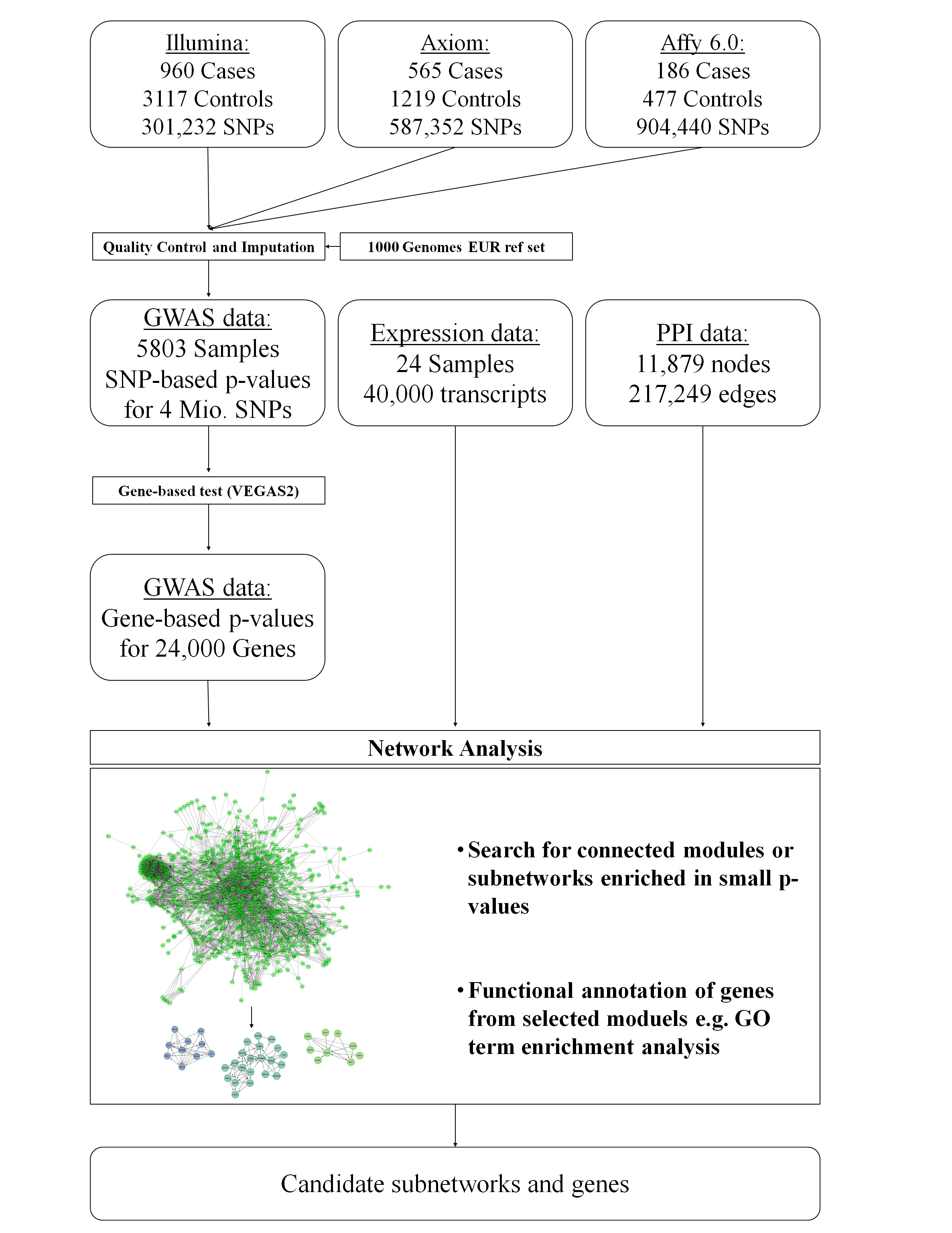

Supplement: S1 Fig — (TIF) [file pone.0158101.s001.tif]
